# Phase-contrast virtual chest radiography

**DOI:** 10.1073/pnas.2210214120

**Published:** 2022-12-29

**Authors:** Ilian Häggmark, Kian Shaker, Sven Nyrén, Bariq Al-Amiry, Ehsan Abadi, William P. Segars, Ehsan Samei, Hans M. Hertz

**Affiliations:** ^a^Department of Applied Physics, KTH Royal Institute of Technology, 114 19, Stockholm, Sweden; ^b^Department of Molecular Medicine and Surgery, Karolinska Institutet, 171 76, Solna, Sweden; ^c^Department of Radiology, Karolinska University Hospital, 171 76, Solna, Sweden; ^d^Department of Clinical Science, Intervention and Technology, Karolinska Institutet, 171 77, Stockholm, Sweden; ^e^Center for Virtual Imaging Trials, Carl E. Ravin Advanced Imaging Laboratories, Department of Radiology, Duke University Medical Center, Durham, NC 27705

**Keywords:** X- ray imaging, phase contrast, chest radiography, virtual clinical trial

## Abstract

Respiratory X-ray imaging enhanced by phase contrast has shown improved airway visualization in animal models. Limitations in current X-ray technology have nevertheless hindered clinical translation, leaving the potential clinical impact an open question. Here, we explore phase-contrast chest radiography in a realistic in silico framework. Specifically, we use preprocessed virtual patients to generate in silico chest radiographs by Fresnel-diffraction simulations of X-ray wave propagation. Following a reader study conducted with clinical radiologists, we predict that phase-contrast edge enhancement will have a negligible impact on improving solitary pulmonary nodule detection (6 to 20 mm). However, edge enhancement of bronchial walls visualizes small airways (< 2 mm), which are invisible in conventional radiography. Our results show that phase-contrast chest radiography could play a future role in observing small-airway obstruction (e.g., relevant for asthma or early-stage chronic obstructive pulmonary disease), which cannot be directly visualized using current clinical methods, thereby motivating the experimental development needed for clinical translation. Finally, we discuss quantitative requirements on distances and X-ray source/detector specifications for clinical implementation of phase-contrast chest radiography.

## Significance

Chest radiography plays an important role in respiratory disease detection, yet the way it is used today is fundamentally limited by the underlying contrast mechanism: X-ray attenuation. This renders subtle pathological changes in the lungs invisible in conventional chest radiography as these do not sufficiently change the overall attenuation through the thorax. The last decades have seen tremendous progress in utilizing the phase shift of X-ray radiation to improve imaging sensitivity. However, human chest imaging with phase contrast remains largely unexplored. In our work, we generate realistic virtual chest radiographs to show that phase-contrast chest radiography can visualize the smallest airways and their disease-related obstruction, which cannot be observed today using the conventional technique.

**Fig. 1.**
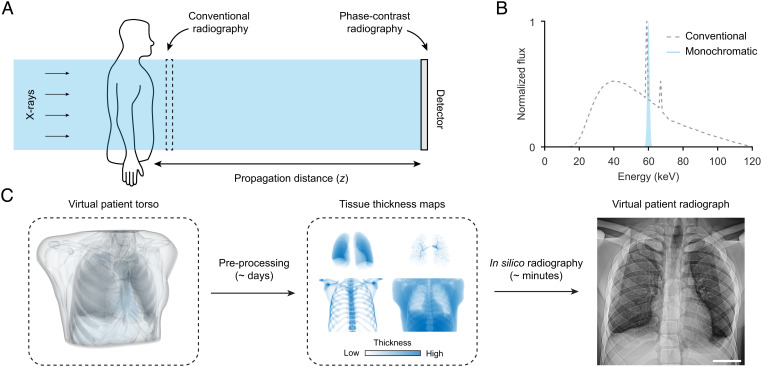
Concept and in silico framework. (*A*) Imaging geometry. The main difference between conventional and phase-contrast chest radiography using PBI is the propagation distance *z*. (*B*) X-ray spectra simulated in this study, with a conventional 120-kVp tungsten spectrum with 4.3-mm Al filtering (dashed) used to simulate conventional radiographs and monochromatic 60 keV (blue) used to simulate phase-contrast radiographs. (*C*) In silico framework for virtual radiography (see details in the *Methods* section). (Scale bar, 5 cm (*C*).)

Phase-contrast techniques improve soft-tissue visualization and thereby the sensitivity of X-ray imaging conventionally based on detecting differential attenuation only (1, 2). Conceptually, these techniques operate by converting the phase shift of transmitted X-rays into observable intensity variations at the detector plane. In the propagation-based imaging (PBI) technique (3), a propagation distance between the imaged object and detector (cf. Fig. 1*A*) enables the detection of interference patterns at the detector arising from Fresnel (i.e., near-field) diffraction in the object. Ultimately, this enhances material interfaces and thus improves the ability to differentiate between tissue types in a biomedical context. The PBI approach has been particularly promising for respiratory imaging as demonstrated preclinically in small-animal models, e.g., mice (4–7), rats (8) and rabbits (9–12) with preliminary studies in larger animals, e.g., pigs (6, 13–15). However, PBI puts stringent requirements on the X-ray source and detector, where imaging of large human torsos would, in principle, currently be possible only at synchrotron facilities. To date, this has yet to be demonstrated due to challenges in conducting clinical trials at these facilities. Parallel to the technological development required for clinical translation to a hospital-scale environment (e.g., compact high-brilliance X-ray sources and high-resolution detectors), the potential clinical impact of respiratory PBI remains largely unknown.

The inherent complexity of clinical studies inhibits early-stage exploratory studies of new clinical imaging techniques. In this context, virtual clinical imaging trials with realistic virtual patients can be used to explore the impact of new techniques long before clinical studies are possible (16). In particular, developments in highly detailed models of the human torso (17) enable realistic simulations of chest X-ray imaging, e.g., radiography or computed tomography (CT). Computational modeling of clinical phase-contrast X-ray imaging is particularly challenging due to the typical few-μm wavefront-sampling requirements for modeling the interference effects in conjunction with the large sizes of human body parts (∼tens of centimeters). To this end, we recently demonstrated a preprocessing strategy unlocking PBI simulations on realistic virtual models of human anatomy (18).

Here, we use our in silico framework for exploring PBI applied to the simplest form of clinical thoracic imaging: chest radiography. This is a challenging computational problem which has not been solved to date because of the large volume of the human thorax (∼30 × 40 × 40 cm) and the high X-ray energies used clinically for sufficient X-ray transmission through the thorax. Chest radiography plays an important role for early detection of pulmonary cancer and other respiratory diseases. Yet, many early-stage

diseases often go undetected in radiography due to anatomical overlap and limited attenuation differences in soft-tissue pathologies. We show through our virtual approach that phase-contrast chest radiography is likely to be ineffective for improving solitary pulmonary nodule detection, yet particularly promising for visualizing small airways.

## Concept and In Silico Framework

We conceptually illustrate the PBI approach to phase-contrast chest radiography in Fig. 1*A*. To isolate the effect of the propagation distance (*z*) on the interference effects observed at the detector plane, we eliminated geometric magnification by modeling a parallel X-ray beam corresponding to a source-to-patient distance ≫*z*. We further investigated the influence on radiograph appearance from a monochromatic beam compared to a conventional tungsten spectrum used in clinical imaging today (Fig. 1*B*). This is particularly relevant since current and emerging high-brilliance X-ray sources, e.g., synchrotron (14) or inverse-Compton (19–22) radiation suitable for PBI at clinical energies can provide narrow-bandwidth X-ray beams.

We developed an in silico framework for generating realistic virtual chest radiographs (Fig. 1*C*). Virtual patients are generated from the highly detailed extended cardiac-torso (XCAT) model originally based on real patient CT data (17, 23). Next, we performed a computationally expensive edge-preserving 3D upsampling of the virtual patients (reducing voxel sizes from 100 μm to sub-10 μm) followed by compression into tissue thickness maps sufficient for generating virtual chest radiographs with conventional or phase-contrast settings. The latter is modeled by numerically evaluating the Fresnel-diffraction integral (18). Lastly, we applied postprocessing on the radiographs using a clinically used commercial software application to further enhance the realism of the virtual radiographs.

## Edge Enhancement vs. Propagation Distance

**Fig. 2.**
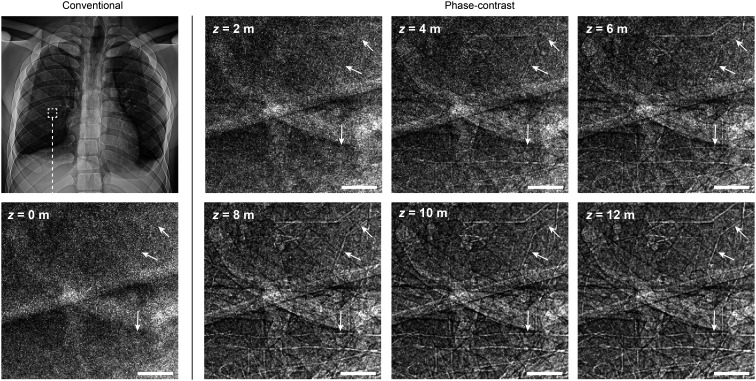
Chest radiograph appearance as a function of propagation distance. Zooming in on a 1 × 1 cm^2^-region of virtual chest radiographs simulated with a 50-μm-pixel idealized photon-counting detector (cf. details in “*Methods*” section). The edge enhancement of tissue interfaces grows stronger with increasing propagation distance *z*, particularly impacting the visibility of small airways (cf. arrows). (Scale bars, 2 mm.)

**Fig. 3.**
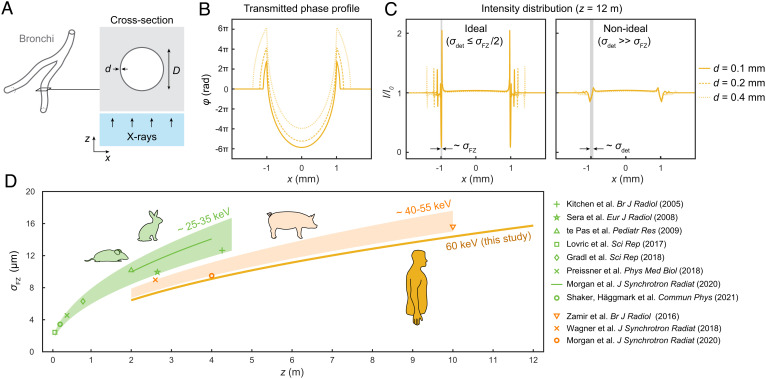
Theoretical Fresnel diffraction in isolated airways. (*A*) Cross-section of an airway with lumen diameter *D* and wall thickness *d* of muscle-like tissue, surrounded by low-density parenchyma. We then model incident X-rays as a plane wave (i.e., corresponding to the parallel beam in our radiographs) propagating in the *z*-direction through the bronchial cross-section. (*B*) Phase-shift (φ) of the 60 keV X-ray wavefront exiting the bronchial cross-section simulated with *D* = 2 mm and varying wall thickness. (*C*) Intensity distribution of transmitted X-rays normalized to the only parenchyma region (*I*/*I*_0_) at *z*= 12 m, with two cases shown (“Ideal” vs. “Non-ideal”) depending on the detector resolution (*σ*_det_) and Fresnel zone width ($\sigma_{\;}\text{FZ}=\sqrt{\lambda z}$). (*D*) Fresnel zone width (*σ*_FZ_) as a function of *z* plotted for X-ray energy ranges suitable for imaging animals with increasing size. Data points from relevant PBI literature on mice, rats, rabbits, and pigs plotted with corresponding references.

The effect of the propagation distance in phase-contrast chest radiography is illustrated in Fig. 2. At a macroscopic level, the chest radiographs appear unchanged with increasing distance (*z* = 2 to 12 m). However, on a submillimeter scale, we observe the expected edge enhancement of tissue boundaries. In particular, we observe that the visibility of small airways (< 2 mm) increases with *z* as the bronchial walls are edge-enhanced. At *z* = 12 m, we measure the width of the edge enhancement to ∼100 μm (2 × 50 μm pixels). This is larger than the theoretical width approximately equal to the first Fresnel zone: $\sigma_{\;}\text{FZ}=\sqrt{\lambda z}$, where *λ* is the X-ray wavelength (24). We illustrate this scenario by studying Fresnel-diffraction in hypothetical isolated bronchi in Fig. 3*A*. The local phase-shift φ of the transmitted X-ray wave-front through the bronchial cross-section (Fig. 3*B*) introduces interference fringes in the transmitted intensity at *z* >  0 (Fig. 3*C*). The fringe amplitudes are proportional to the local phase gradient |∇φ|, which is maximized at the air/wall interface (negligibly influenced by the wall thickness). Assuming perfect spatial coherence of the incident X-rays (e.g., the parallel beam simulated in our chest radiographs), the fringe visibility or contrast is determined by the spatial resolution of the detector *σ*_det_ in relation to *σ*_FZ_ (cf. Fig. 3*C*). The virtual detector in our in silico radiography situation has *σ*_det_ = 100 μm, which is larger than *σ*_FZ_ ≈ 16 μm at 60 keV and *z* = 12 m, indicating that the visibility and apparent width of the fringes in all of our phase-contrast chest radiographs are detector-limited. It is worth noting that the optimal fringe visibility is achieved when *σ*_det_ ≤ *σ*_FZ_/2, e.g., for ideal Nyquist-sampling of the first Fresnel-zone (cf. “Ideal” case in Fig. 3*C*), although a larger *σ*_det_ can still be sufficient for observing the edge enhancement at lower contrast (cf. “Non-ideal” case in Fig. 3*C*). Lastly, we note that phase-contrast imaging in the near-field regime requires much longer *z* at clinical energies for Fresnel zone widths similar to those achieved in PBI of animal models at lower energies (Fig. 3*D*).

## Detecting Solitary Pulmonary Nodules

**Fig. 4.**
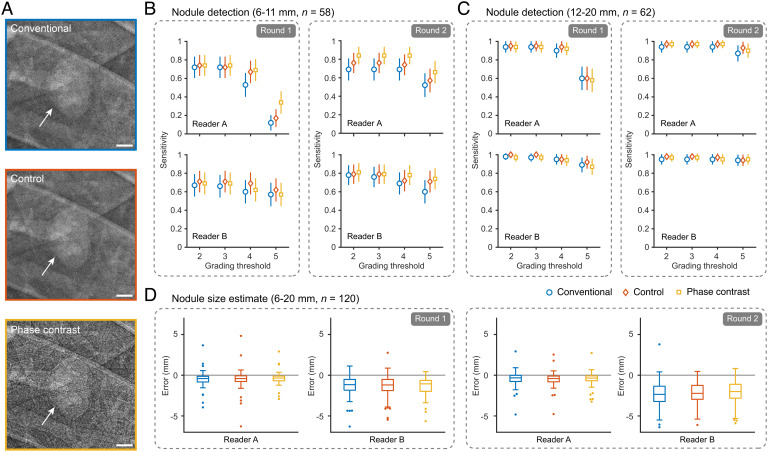
Edge enhancement does not improve pulmonary nodule detection. (*A*) Example of a pulmonary nodule (cf. arrows) inserted into the virtual patient appearing in chest radiographs simulated with the different settings shown here for a 2 × 2-cm^2^ area. (*B*) Detection sensitivity for smaller nodules (6 to 11 mm) with error bars corresponding to 95% CI. (*C*) Detection sensitivity for larger nodules (12 to 20 mm); error bars correspond to 95% CI. (*D*) Errors in size estimates of all nodules (6 to 20 mm). Outliers outside box plots are represented as dots. (Scale bars, 2.5 mm (*A*).) Raw data in (*B*) and (*C*) can be found in *SI Appendix*, Table S1 and S2, respectively.

The first realistic clinical application of phase-contrast chest radiography we explored was for detecting solitary pulmonary nodules (6 to 20 mm) where early-stage detection is imperative for reducing lung cancer mortality (25). Although CT has higher nodule detection sensitivity than radiography, improved radiographic screening could be a more cost-effective and lower-dose alternative to CT-screening proposals (26). To this end, we created a dataset from virtual chest radiographs consisting of 80 unique 8 × 8 cm^2^-region of interests (ROIs) containing 0 to 3 nodules each. Each unique ROI was then simulated with three different settings: conventional, control, and phase contrast for a total of 240 ROIs (see details in *Methods* section). Single-blinded reader study with two clinical radiologists who were instructed to look for nodules in all randomly presented ROIs, and they graded their degree of suspicion on a five-point scale (1, no nodule; 5, certain nodule). The readings were performed in two rounds spaced by 4 wk, including a session 2 wk after the first round where the radiologists were trained in reading phase-contrast chest radiographs. By analyzing the results, we found no statistically significant improvement neither in detection sensitivity nor in size estimates of the pulmonary nodules when comparing ROIs from phase-contrast and conventional chest radiographs (cf. Fig. 4). This was true both before and after the training session (cf. “Round 1 & 2” in Fig. 4*B*–*D*). We also note that the different spectra (60-keV monochromatic versus 120-kVp tungsten anode) had no qualitative influence on conventional chest radiograph appearance according to the radiologists and no quantitative influence on nodule detection (cf. Fig. 4, “Control”).

## Visualizing Small-Airway Obstruction

**Fig. 5.**
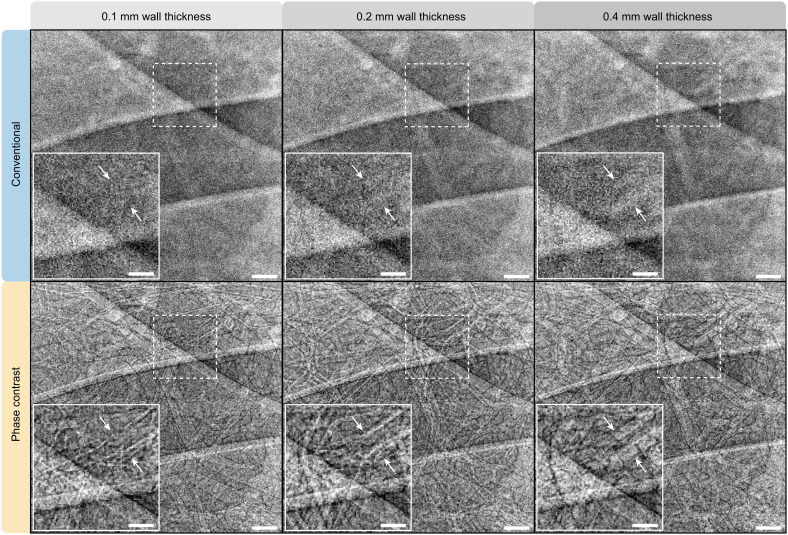
Visualizing wall thickening of small airways. Here, a smaller area (2 × 2 cm^2^) from a virtual chest radiograph is shown (*Top*, conventional; *Bottom*, phase contrast at *z* = 12 m) with increasingly thicker airway walls: 0.1 mm, 0.2 mm, and 0.4 mm. The insets (*Bottom Left*) of each panel are zoom-ins of an airway (indicated by the arrows) with the walls clearly visualized in the phase-contrast radiograph. The pixel size in all images is 50 μm. (Scale bars, 2 mm (overview), 1 mm (*inset*).)

**Fig. 6.**
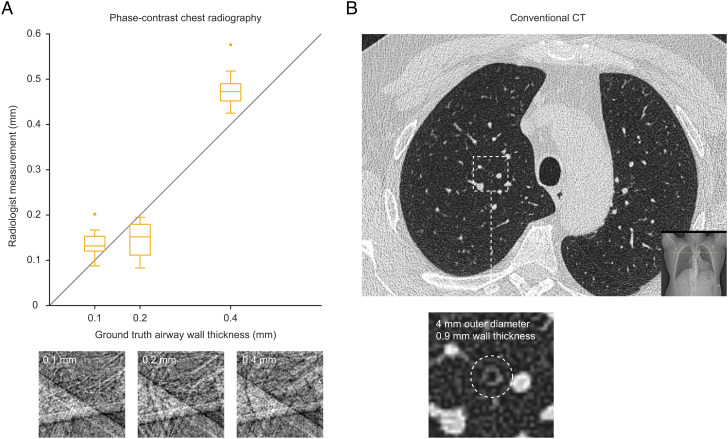
Quantifying small-airway obstruction. (*A*) Four radiologists were tasked to measure the airway wall thickness at 0.1-mm, 0.2-mm, and 0.4-mm airway wall thickness. Box plots (*n* = 10 measurements each) corresponding to their measurements shown for each degree of obstruction. Dots represent outliers. (*B*) Axial CT images from a healthy 56-y-old male with subjective breathing problems, performed on a state-of-the-art Siemens Somatom Drive (Siemens, Munich, Germany). CT parameters were 0.5-mm slice thickness, 372-mm field-of-view (lung kernel Br59f reconstruction) resulting in a 0.7-mm pixel size. Bronchi down to 4 mm and wall thicknesses down to the pixel size can be observed in the CT dataset.

Encouraged by the improved airway visualization over conventional radiography (cf. Figs. 2 and 3), we explored the potential clinical application of assessing small-airway obstruction in radiographs. To this end, we modified local volumes of the virtual patient lungs to model thicker airway walls and found that the wall thickening could be clearly distinguished in phase-contrast chest radiography (cf. lower row in Fig. 5). This further enabled a quantitative assessment of the wall thicknesses by four clinical radiologists directly on the radiographs (cf. Fig. 6*A*) with measurement errors mainly limited by the virtual detector resolution. We note that even with our idealized detector with higher resolution than clinically used detectors today (typically ≥200 μm), attenuation contrast alone in conventional radiography (cf. top row in Fig. 5) is clearly insufficient for observing obstruction in these small airways. Although bronchial walls can be observed in conventional clinical attenuation-based CT, the measurement accuracy is insufficient for quantifying small-airway obstruction (cf. Fig. 6*B*).

## Discussion

This work represents a theoretical exploration of phase-contrast chest radiography. Specifically, we show that our virtual framework enables realistic explorations, which are particularly relevant as the experimental realization of clinical phase-contrast X-ray imaging is currently in its infancy. By overcoming the computational challenges associated with μm-level sampling of virtual patients required for Fresnel-diffraction modeling of X-ray wave propagation, we could generate virtual chest radiographs perceived as realistic enough by our radiologists for predicting the potential diagnostic impact of phase-contrast chest radiography. Here, we briefly summarize the key results and discuss quantitative source and detector requirements for clinical implementation.

In a reader study focusing on solitary pulmonary nodules, we found that the phase-contrast enhancement of nodule borders resulted in no statistically significant improvement in nodule detection rate and size estimates. This is likely due to the solid nodules presenting sufficient attenuation contrast in the low-density lung parenchyma background in addition to the edge enhancement being unspecific. We anticipate, however, that the edge enhancement could be advantageous for deciphering small details such as nodule spiculation and for differentiating nodule types, a potential that we did not explore in the present study.

Instead, we predict that the main impact of phase-contrast chest radiography will be for visualizing small airways. The edge enhancement arising from near-field diffraction in the small airways is more challenging to detect at these clinical X-ray energies (∼60 keV) compared to preclinical imaging scenarios where most PBI has been performed to date (cf. Fig. 3*D*). We note that obstruction in the small airways is central in the pathophysiology of a number of common diseases (e.g., COPD and asthma), where wall thickening of bronchi less than 2 mm in diameter is believed to be responsible (27, 28). In the clinical setting today, the latter cannot be directly observed in vivo, and current measurements are therefore indirect, e.g., impulse oscillometry (29, 30) and nitrogen washout (31). In light of this, phase-contrast chest radiography has the potential to become the first technique for directly visualizing wall thickening in small-airway disease in vivo. We also show the potential for manual quantification of the airway wall thickening directly on 2D images (cf. Fig. 6), possibly providing a path toward an automated prediction of small-airway obstruction in whole lungs using computer-assisted algorithms applied to the radiographs, e.g., similar to AI-assisted lung cancer predictions from CT images (32).

Low-frequency background contribution from Compton scattering is a central problem in all forms of chest X-ray imaging. In our study, we deliberately assumed an ideal scatter rejection scheme where no Compton-scattered photons reached the detector. The rationale behind this choice was that incorporating a *z*-dependent Compton-scattering background (∝ <  *SPSDOUBLEDOLLAR* >  1/*z*^2^ for multiple-scattering events) would make it difficult to compare conventional and phase-contrast chest radiographs acquired at different *z*. Nevertheless, we note that background scattering is a nonissue at patient-to-detector distances of a few meters and beyond (cf. *SI Appendix*, Fig. S4), which is consistent with the well-known use of air gap techniques (33) as alternatives to antiscatter grids.

Although our rationale for exploring phase-contrast chest radiography at 60 keV was its equivalence to the weighted average of a conventional 120-kVp tungsten anode spectrum (i.e., resulting in similar transmission through the patient), we note that the optimal phase-contrast energy is highly dependent on patient size and imaging task. For thinner patients like the one in our study, we note that airway visibility is likely optimal at lower energies (40 to 60 keV, cf. *SI Appendix*, Fig. S3) where both Fresnel-zone widths and X-ray refractive indices are larger. For thicker patients, the optimal X-ray energy will naturally increase to ensure sufficient photon statistics at the detector due to lower patient transmission. Future studies should therefore explore optimal energies for phase-contrast chest radiography in a wider virtual patient population and specific imaging tasks (e.g., airway or nodule visualization).

The in silico framework presented here has its limitations. Further virtual explorations of phase-contrast chest radiography for diagnosis of other common respiratory conditions (e.g., emphysema, pneumonia) across a wider virtual patient cohort requires improving the preprocessing time per virtual patient (currently ∼14 d; see details in “*Methods*” section). We note that the upsampling component was suboptimally implemented in this study and that ongoing optimizations aim to reduce the preprocessing time to < 1 d per virtual patient. Another limitation of current state-of-the-art virtual models of the human torso is that they do not model anatomy on the alveolar level, where the specific contribution of individual alveoli to background speckle noise (34) needs to be evaluated in realistic clinical settings.

While the clinical translation of PBI is challenging, we have demonstrated here that phase-contrast chest radiography could have a clinical relevance motivating further experimental development. We note that the related technique—dark-field imaging—is an example of successful translation from concept to motivating preclinical results and recent clinical demonstrations (35, 36) and extension to CT (37). On the X-ray source side, although we have simulated a source with perfect spatial coherence, the requirement for phase-contrast chest radiography is that the X-ray illumination on the patient must be spatially coherent over the first Fresnel zone (*σ*_FZ_). In mathematical terms, the transverse coherence length *l*_t_ ≈ *Rλ*/*σ*_src_ should ideally be larger than $\sigma_{\;}\text{FZ}/\sqrt{2}=\sqrt{\lambda z/2}$, where *R* is the source-to-patient distance and *σ*_src_ is the spatial extent of the source (38). In numerical terms, assuming 60-keV X-rays and *z* = 6 m yields the ratio $R/\sigma_{\;}\text{src}>\sqrt{z/(2\lambda )}\approx 3.8\cdot 10^{5}$. For a hypothetical source with *σ*_src_ = 50 μm, this indicates a required *R*≥ 20 m. As we can no longer assume that *R* >  > *z* (i.e., the parallel beam modeled in this study), the introduced magnification *M* reduces the effective propagation distance by a factor 1/*M*. To illustrate, *R*= 20 m indicates a required patient-to-detector distance of ≈8.6 m (corresponding to *z* = 6 m and *M*= 1.43), which ultimately leads to a total system size of < 30 m (source-to-detector distance). Here, we note that emerging inverse-Compton scattering sources could be a candidate for fulfilling these requirements. Prototypes currently being developed at both research institutions (20–22) and commercially (19) aim at energy ranges suitable for clinical radiography.

On the detector side, improved contrast of the Fresnel fringes in the 5 to 15 μm-range (cf. Fig. 3*D*) could be achieved with subpixel localization schemes with photon-counting detectors reaching few-μm spatial resolution (39). Recently demonstrated stretchable high-resolution scintillators (40) are also promising alternatives to conventional flat-panel or upcoming photon-counting detectors for large field-of-view applications such as chest radiography. We note that improved detector resolution can also be used to shorten the distances in PBI (i.e., *R* and *z*) while still resolving the edge enhancement.

Lastly, the extension of clinical PBI from radiography to CT holds promise for visualizing the whole tracheobronchial tree down to the few-millimeter small airways and should therefore be a key focus of further virtual explorations.

## Materials and Methods

### Virtual Patients.

A virtual patient (female, 57.5 kg weight, 170 cm height) was generated from the extended cardiac–torso (XCAT) model (23), which is a highly detailed anatomical model based on patient CT data. The patient model included intraorgan heterogeneities in the lungs and bone (17, 41). Specifically, the lungs contained pulmonary vasculature, airways, and lung parenchyma texture at a 0.1-mm resolution. The tracheobronchial tree was generated down to 0.25-mm diameter airways. Of note, 12 unique tissue material compositions were included in the model, some of which were further varied in density to model intraorgan heterogeneity (e.g., the lung parenchyma and bone texture; *SI Appendix*, Table S4 and Fig. S2).

To enable virtual phase-contrast imaging, the virtual patient had to be upsampled to comply with the high sampling requirements of wave-propagation simulations: $x_{s}\leq \sigma_{\;}\text{FZ}/2=\sqrt{\lambda z}/2$, where *x*_*s*_ is the numerical sampling, *λ* the X-ray wavelength, and *z* the propagation distance. The model was upsampled to ∼3-8-μm resolution corresponding to *z* = 2 to 12 m according to ref. (18). Preprocessing was performed in MATLAB on a computer node with 1 TB RAM and 4×12 CPU cores Intel E7-8857v2 Ivy Bridge. The total preprocessing time for the virtual patient was 14 d.

### In Silico Chest Radiography.

We simulated conventional and phase-contrast chest radiographs (cf. Fig. 1) of the processed virtual patient using a computational wave-propagation approach (18). Three different settings were simulated:

(i) Phase-contrast chest radiography, simulated with a monochromatic beam (60 keV) at *z*= 2 to 12 m.
(ii) Conventional chest radiography, simulated with a 120-kVp tungsten spectrum (with 4.3-mm aluminum filtration (42)) at *z* = 0 m.
(iii) Control radiography, simulated with a monochromatic beam (60 keV) at *z* = 0 m.

The monochromatic spectrum was chosen in (i) as coherent sources suitable for propagation-based phase-contrast X-ray imaging available today are either synchrotrons (14) or inverse-Compton scattering sources (19–22) both of which provide monochromatic beams (60 keV is the average energy of the conventional tungsten spectrum, cf. Fig. 1*B*).

The radiographs simulated with settings (i) to (iii) all correspond to 0.1-mSv effective dose, calibrated using Monte Carlo simulations (MC-GPU (43)) on the same virtual patient. This dose corresponds to incident X-ray fluxes on the virtual patient of 3.6 ⋅ 10^6^ X-ray photons/mm^2^ (60 keV monochromatic) and 4 ⋅ 10^6^ photons/mm^2^ (120 kVp tungsten spectrum). We note that these fluxes would correspond to a submillisecond exposure time at suitable synchrotron beamlines, e.g., IMBL at the Australian Synchrotron, where flux is > 10^10^ photons/mm^2^/s at suitable filtration (44). For all radiographs, we simulated a parallel beam corresponding to a long source-to-patient distance *R* (i.e., synchrotron or inverse-Compton scattering sources).

For the detector, we modeled a CdTe photon-counting detector with 50-μm pixels, 0.75-mm thick sensor, and single-pixel point-spread function. We applied postprocessing to the simulated radiographs with a vendor-specific processing software application for chest radiography (Siemens AG). Although the software is a black box, it likely performs a combination of standard processing steps including spatial frequency filtering (e.g., unsharp mask filtering), dynamic range reduction, and denoising (45). Nevertheless, we acknowledge that using a vendor-specific proprietary algorithm creates potential reproducibility challenges (46).

### Fresnel Diffraction in a Theoretical Airway.

In Fig. 3, we modeled an isolated airway as an air-filled cylinder (0.0012 g/cm^3^ density) with lumen diameter *D* and wall of thickness *d* of homogenous muscle tissue (1.05 g/cm^3^ density) surrounded by a homogenously modeled lung parenchyma (0.53 g/cm^3^ density). We then set *D* = 2 mm with varying *d* (0.1 to 0.4 mm) and then calculated the phase shift φ according to φ = −2*π*/*λ* ⋅ ∫*δ*(*λ*) d*z*, where *z* is the direction of X-ray propagation and *δ*(*λ*) is the material-dependent decrement from unity of the real part of the X-ray refractive index (Re{*n*(*λ*)} = 1 − *δ*(*λ*)). For 60 keV X-rays (→*λ* ≈ 0.02 nm), we used the *δ*(*λ*) values 6.91 ⋅ 10^−11^, 6.66 ⋅ 10^−8^, and 3.37 ⋅ 10^−8^ for air, muscle, and lung parenchyma tissue, respectively (tissue composition from ref. (47)).

### Reader Study on Nodule Detection.

We created a dataset of 80 unique ROIs (8 × 8 cm^2^ each) from simulated chest radiographs of the virtual patient, with each ROI containing 0 to 3 solitary pulmonary nodules inserted into random locations of the lung parenchyma. We modeled solid noncalcified pulmonary nodules as these are more challenging to detect than calcified equivalents. The nodule tissue density was set to a muscle tissue equivalent (48). Sizes of nodules ranged from smaller (6 to 11 mm, *n* = 58) to larger (12 to 20 mm, *n* = 62) for a total of 120 nodules in the dataset. As current recommendations are that only nodules > 5 mm require follow-up, we included only nodules 6 mm or larger (49). We simulated the dataset with the three different settings (i) to (iii) for a total of 80×3 = 240 ROIs containing 120×3 = 360 nodules. For phase-contrast radiographs, we simulated a long propagation distance (*z* = 12 m) for a strong edge-enhancement effect.

Two readers (active radiologists, S.N. and B.A., with 30 and 14 y of experience, respectively) individually reviewed the complete dataset presented in random order. Prior to the study, the readers had no experience with studying phase-contrast chest radiographs. The impact of training was explored by performing complete readings in two rounds: once 2 wk before a training session in the characteristics of phase-contrast radiography (“Round 1”) and once 2 wk after the training session (“Round 2”). For each ROI, the readers were instructed to locate potential nodules and grade their level of confidence on a five-point scale (1, no nodule; 5, certain nodule) as well as measuring the nodule size. Evaluations were performed on PACS end stations. True-positives (TP), false-positives (FP), and false-negatives (FN) were extracted from the readings. Nodule detection sensitivity values were calculated with a 95% two-sided CI.

### Visualization of Airway Wall Thickening.

A local lung volume in the virtual patient was modified to simulate three different stages of wall thickening: We simulated airway walls with 0.1 mm, 0.2 mm, and 0.4 mm thickness. We then studied the appearance of these airways when imaged with conventional and phase-contrast chest radiography (with *z* = 12 m). Four active radiologists (30, 30, 7, and 5 y of experience, respectively) quantified the wall thickness directly on phase-contrast radiographs with three measurements each on different airways.

## Supplementary Material

Appendix 01 (PDF)

## Data Availability

Data underlying this study can be found in the article, the *SI Appendix*, and the corresponding Zenodo repository, https://doi.org/10.5281/zenodo.7030749 (50).
