# Supplementary material for: Phase-contrast virtual chest radiography
Source: Proc Natl Acad Sci U S A. 2022 Dec 29;120(1):e2210214120. doi: 10.1073/pnas.2210214120 (PMC9910502; doi:10.1073/pnas.2210214120)
Supplement: Supplementary file 1 — Appendix 01 (PDF) [file pnas.2210214120.sapp.pdf]

PNAS

Supplementary Information for
Phase-contrast virtual chest radiography

Ilian Häggmark*, Kian Shaker*, Sven Nyrén, Bariq Al-Amiry, Ehsan Abadi, William P. Segars, Ehsan Samei, and Hans M. Hertz

* Equal contributors

Corresponding authors: Ilian Häggmark, Kian Shaker
Email: ilian.haggmark@biox.kth.se, kiansd@kth.se

This PDF file includes:

Figures S1 to S4
Tables S1 to S4
SI References

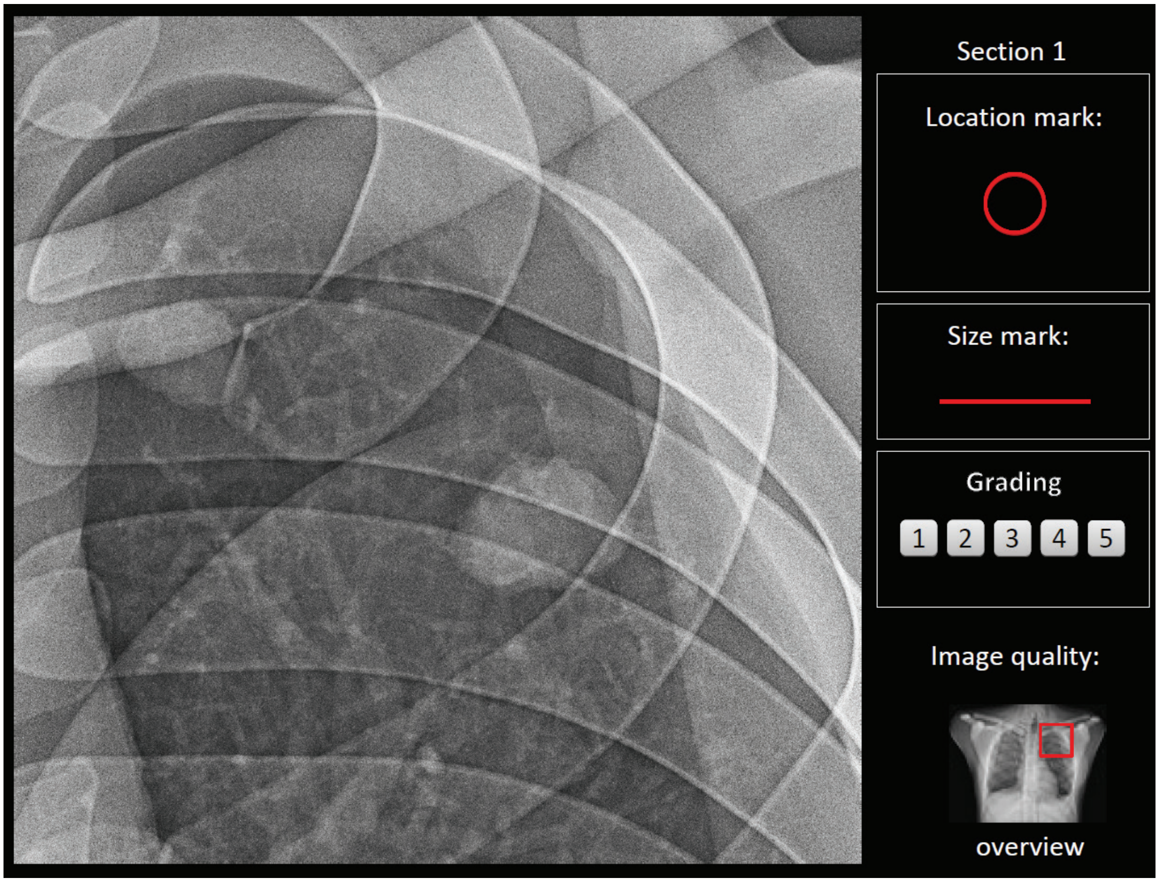

Fig. S1. Example of simulated conventional chest radiograph ROI used in reader study. A PowerPoint file with one ROI per slide was given to the readers for reviewing all ROIs and grade suspicious pulmonary nodules. The instruction on each slide was to place the two red shapes found on the right-hand side on suspected nodules along with a grading label (1-5). The circular shape ("Location mark") was used for evaluating if the readers found the nodules, and the line ("Size mark") was used to calculate the size estimated by the radiologists. Perceived image quality was reviewed by moving a "Grading" label to the "Image quality" field. Prior to reviewing the 240 ROIs used in the study, the PowerPoint includes a test set of 20 ROIs was used to make the readers familiar with the material and instructions. This test set was not included when analyzing the results found in the main manuscript.

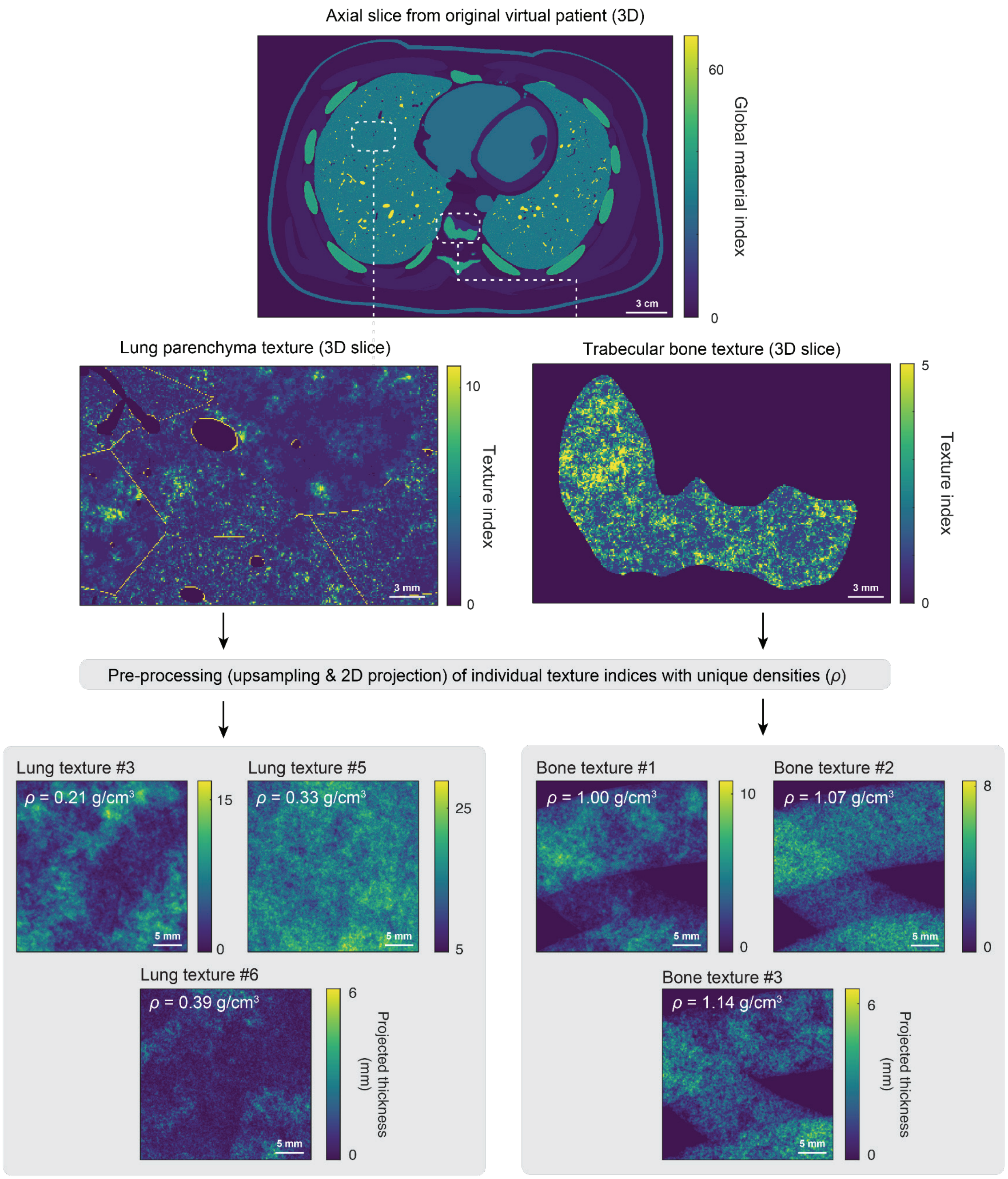

Fig. S2. Intra-organ bone and lung heterogeneity in the virtual patient. The virtual patient, derived from the XCAT model, contains textured heterogeneity in the lungs and bones. These textures are modeled as materials with the same atomic composition but with varying density (ρ). The pre-processing procedure of the virtual patient (i.e., upsampling and 2D projection) necessary for simulating phase-contrast chest radiography preserves these textures, as shown in the bottom part of the figure for a few examples of texture indices in bones (right) and lungs (left) all simultaneously present in the virtual patient.

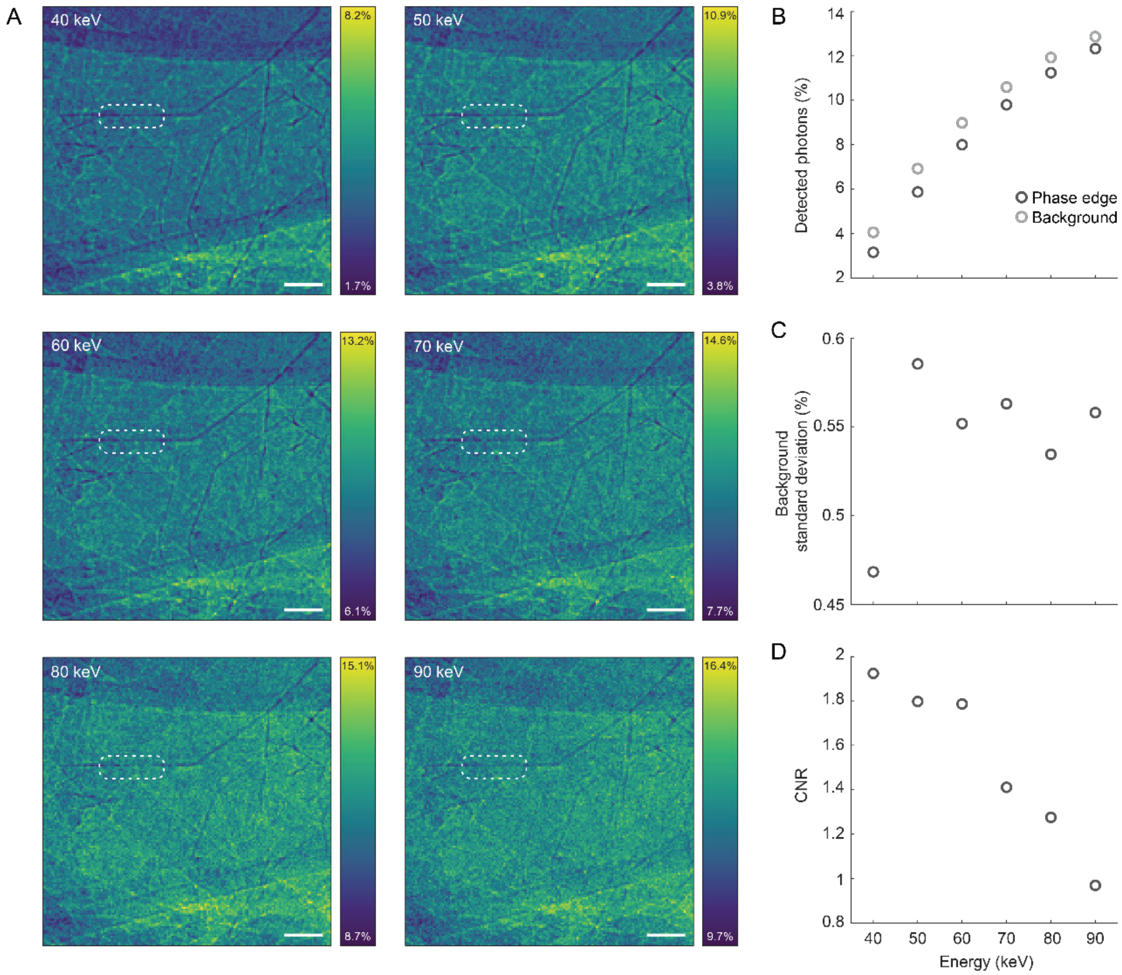

Fig. S3. Airway visibility as a function of X-ray energy in phase-contrast chest radiography. **A**, Local region of the virtual patient lungs imaged with X-ray energies of varying energy (40-90 keV) where the colormap corresponds to the fraction of photons being captured by the detector at z = 12 m. For fair comparison between X-ray energies, the dose to the patient was set to 0.1 mSv in all images and the CdTe sensor thickness was set to 2 mm to ensure a uniform detector efficiency (>90%) over the 40-90 keV energy range. The visibility of the wall of a small airway (dashed box) was then quantified. **B**, Fraction of transmitted photons reaching the detector at the phase edge of the airway compared to the background transmission taken as pixel-rows above and below the phase-edge. **C**, Standard deviation of the background transmitted photon fraction as a function of X-ray energy. **D**, Contrast-to-noise ratio (CNR) for the phase edge of the airway wall as a function of X-ray energy. Scale-bars: 1 mm (A)

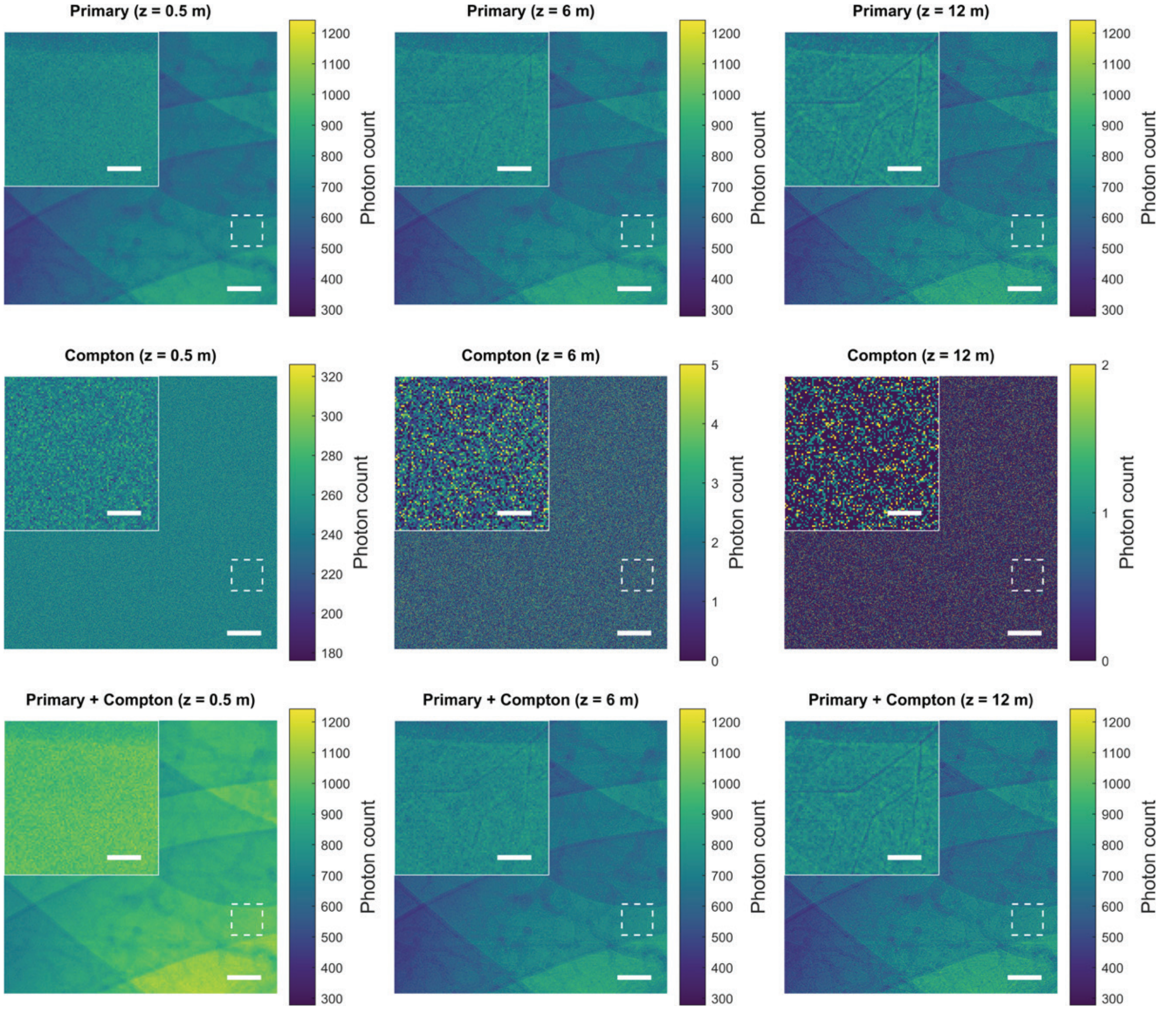

Fig. S4. Compton scattering contributes negligibly at long propagation distances. We evaluate the chest radiograph contribution from primary (i.e., non-interacting transmitted photons) and Compton-scattered photons at increasing propagation distances (left to right, z = 0.5, 6, 12 m). Compton scattering is the dominant interaction in the virtual patient thorax accounting for >85% of the interactions with a mean free path (MFP) of 5.7 cm compared to the photoelectric MFP of 73 cm at 60 keV in soft tissue (~1 g/cm^3). The average soft tissue density-normalized thickness of our virtual patient is ~12.5 cm indicating that multiple Compton scattering of incident X-ray photons dominates over single scattering events, resulting in a near-isotropic angular scattering distribution. Consequently, scattering contribution decreases ($\propto 1/z^2$, cf. middle row) compared to primary photons alone (cf. top row) at increasing *z*. Taken together (cf. bottom row) it becomes clear that Compton scattering is a non-issue at longer *z* and has negligible influence on observing the edge enhancement of airways. Scale bars: 5 mm (overview), 1 mm (zoom-in).

Table S1. Detection sensitivities for smaller nodules (6-11 mm)

		Smaller nodules (6-11 mm, n = 58)					
		Sensitivity before training (%)			Sensitivity after training (%)		
**Reader**	**Confidence**	**Convent.**	**Control**	**Phase-cont.**	**Convent.**	**Control**	**Phase-cont.**
**A**	**Probably no nodule (grading 2+3+4+5)**	**72** (61, 84)	**74** (63, 85)	**74** (63, 85)	**69** (57, 81)	**76** (65, 87)	**84** (75, 94)
	**Uncertain (grading: 3+4+5)**	**72** (61, 84)	**72** (61, 84)	**74** (63, 85)	**69** (57, 81)	**76** (65, 87)	**84** (75, 94)
	**Probably nodule present (grading: 4+5)**	**53** (41, 66)	**67** (55, 79)	**69** (57, 81)	**69** (57, 81)	**74** (63, 85)	**84** (75, 94)
	**Definitely nodule present (grading: 5)**	**12** (4, 20)	**17** (8, 27)	**34** (22, 47)	**52** (39, 65)	**57** (44, 70)	**66** (53, 78)
**B**	**Probably no nodule (grading 2+3+4+5)**	**67** (55, 79)	**71** (59, 82)	**69** (57, 81)	**78** (67, 88)	**79** (69, 90)	**81** (71, 91)
	**Uncertain (grading: 3+4+5)**	**66** (53, 78)	**71** (59, 82)	**69** (57, 81)	**76** (65, 87)	**79** (69, 90)	**81** (71, 91)
	**Probably nodule present (grading: 4+5)**	**60** (48, 73)	**69** (57, 81)	**62** (50, 75)	**69** (57, 81)	**72** (61, 84)	**78** (67, 88)
	**Definitely nodule present (grading: 5)**	**57** (44, 70)	**62** (50, 75)	**57** (44, 70)	**60** (48, 73)	**71** (59, 82)	**74** (63, 85)

Data above shown for conventional, control and phase-contrast ROIs. Values in parenthesis correspond to the 95% two-sided confidence intervals.

Table S2. Detection sensitivities for larger nodules (12-20 mm)

		Larger nodules (12-20 mm, n = 62)					
		Sensitivity before training (%)			Sensitivity after training (%)		
**Reader**	**Confidence**	**Convent.**	**Control**	**Phase-cont.**	**Convent.**	**Control**	**Phase-cont.**
**A**	**Probably no nodule (grading 2+3+4+5)**	**94** (87, 100)	**95** (90, 100)	**94** (87, 100)	**94** (87, 100)	**97** (92, 100)	**97** (92, 100)
	**Uncertain (grading: 3+4+5)**	**94** (87, 100)	**95** (90, 100)	**94** (87, 100)	**94** (87, 100)	**97** (92, 100)	**97** (92, 100)
	**Probably nodule present (grading: 4+5)**	**90** (83, 98)	**94** (87, 100)	**92** (85, 99)	**94** (87, 100)	**97** (92, 100)	**97** (92, 100)
	**Definitely nodule present (grading: 5)**	**60** (47, 72)	**60** (47, 72)	**58** (46, 70)	**87** (79, 95)	**93** (87, 100)	**90** (83, 98)
**B**	**Probably no nodule (grading 2+3+4+5)**	**98** (95, 100)	**100**	**97** (92, 100)	**95** (90, 100)	**98** (95, 100)	**97** (92, 100)
	**Uncertain (grading: 3+4+5)**	**97** (92, 100)	**100**	**97** (92, 100)	**95** (90, 100)	**98** (95, 100)	**97** (92, 100)
	**Probably nodule present (grading: 4+5)**	**95** (90, 100)	**95** (90, 100)	**94** (87, 100)	**95** (90, 100)	**97** (90, 100)	**95** (90, 100)
	**Definitely nodule present (grading: 5)**	**89** (81, 97)	**92** (85, 99)	**87** (79, 95)	**94** (87, 100)	**94** (87, 100)	**95** (90, 100)

Data above shown for conventional, control and phase-contrast ROIs. Values in parenthesis correspond to the 95% two-sided confidence intervals.

Table S3. True-positives (TP) and false-positives (FP) from the nodule detection study.

	Before training				After training				
	Conventional		Phase-contrast		Conventional		Phase-contrast		
**Reader**	**TP**	**FP**	**TP**	**FP**	**TP**	**FP**	**TP**	**FP**	**CP**
**A**	87	3	97	4	98	5	109	5	120
**B**	94	2	94	5	99	9	104	13	120
**A+B**	181	5	191	9	197	14	213	18	240

Data above shown for conventional and phase-contrast ROIs. Values presented here correspond to nodules graded with either 4 or 5 (corresponding to “probable“ and “certain” confidence of nodule presence). The right-most column (condition positives, CP = TP + FP) shows the total number of nodules in the corresponding datasets. Table values correspond to nodules of all sizes.

Table S4. Tissue materials and densities used for the virtual patient generated from XCAT model.

**Tissue material**	**Density** ρ **(g/cm**$^{3)}$
Air	0.0013
Adipose tissue	0.92
Blood (Arteries/Veins)	1.06
Cartilage	1.10
Lung parenchyma (textured)	0.10, 0.15, 0.21, 0.27, 0.33, 0.39, 0.45, 0.53, 0.61, 0.69, 0.77, 0.85, 0.92
Muscle tissue	1.05
Ribs	1.92
Skin	1.09
Soft tissue	1.05
Sternum	1.08
Bone (Spine/Pelvis)	1.38
Trabecular bone texture	1.00, 1.07, 1.14, 1.21, 1.28

The 12 unique tissue material compositions above were included in the virtual patient phantom. These were assigned the densities above from the original XCAT model, we then used elemental composition of each material from ITIS [1] and X-ray transmission properties from ref. [2]. Pulmonary nodules were assumed to have similar composition as muscle tissue.

SI References

1. IT'IS Foundation, Tissue Properties Database V4.0 2018, doi: 10.13099/VIP21000-04-0

2. Henke BL, Gullikson EM, and Davis JC. X-ray interactions: photoabsorption, scattering, transmission, and reflection at e=50-30000 ev, z=1-92. At. Data Nucl. Data Tables 1993;54:181-342. doi: 10.1006/adnd.1993.1013
